# CLINICAL OUTCOMES OF ACL RECONSTRUCTION WITH THREE DIFFERENT AUTOGRAFTS

**DOI:** 10.1590/1413-785220263405e305203

**Published:** 2026-08-10

**Authors:** Thiago Castor Cesar, Matheus Ornellas Costa, Luiz Felipe Mundim de Souza, Moisés de Toledo Vilela, Lucas Galuppo Fernandes Félix, Guilherme Barbosa Moreira

**Affiliations:** 1 Hospital Universitario Ciencias Medicas, Belo Horizonte, MG, Brazil.

**Keywords:** Anterior Cruciate Ligament, Anterior Cruciate Ligament Reconstruction, Knee, Patellar Ligament, Tendons, Quadriceps Muscle, Ligamento Cruzado Anterior, Reconstrução do Ligamento Cruzado Anterior, Joelho, Ligamento Patelar, Tendões, Músculo Quadríceps

## Abstract

**Objective:**

To compare short-term clinical and functional outcomes (0–6 months) of Anterior Cruciate Ligament (ACL) reconstruction using rectus femoris autograft versus established graft options.

**Methods:**

Prospective cohort study (Level II) including 35 patients who underwent ACL reconstruction between 2024 and 2025. Pain, Lysholm score, and range of motion (ROM) were assessed preoperatively, on postoperative day (POD) 1, POD 15, and at six months. Statistical analysis included ANOVA, Kruskal-Wallis tests, and linear mixed-effects models (α = 0.05).

**Results:**

The sample (15 hamstring, 11 rectus femoris, and 9 patellar tendon grafts) was homogeneous (p < 0.05). Significant improvements over time were observed in Lysholm scores (p < 0.001) and knee flexion ROM (p < 0.001), with no influence of graft type (p = 0.167 and p = 0.177, respectively). On POD 1, pain scores were similar among groups (p = 0.701), with mean Visual Analog Scale (VAS) values of 2.20 (hamstrings), 2.18 (rectus femoris), and 1.78 (patellar tendon). On POD 15, mean pain scores were 1.27, 1.73, and 3.00, respectively, remaining statistically comparable (p = 0.149).

**Conclusion:**

ACL reconstruction using the rectus femoris autograft demonstrates clinical and functional outcomes equivalent to patellar tendon and hamstring grafts within the first six months postoperatively. ***Level of Evidence II; Prospective cohort study.***

## RESUMO

**Objetivo:**

Comparar resultados clínico-funcionais (0–6 meses) da reconstrução do Ligamento Cruzado Anterior (LCA) utilizando autoenxerto do reto femoral em relação aos enxertos já consagrados.

**Métodos:**

Estudo de coorte prospectivo (Nível II) com 35 pacientes operados entre 2024–2025. Avaliaram-se dor, escala de Lysholm e amplitude de movimento (ADM) no pré-operatório, 1º e 15º dias pós-operatórios (DPO) e após seis meses. A análise incluiu testes ANOVA, Kruskal-Wallis e modelos lineares mistos (α = 0,05).

**Resultados:**

A amostra (15 flexores, 11 reto femoral, 9 patelar) foi homogênea (p > 0,05). Observou-se melhora significativa na Lysholm (p < 0,001) e ADM de flexão (p < 0,001) ao longo do tempo, sem influência do tipo de enxerto (p = 0,167 e p = 0,177, respectivamente). No 1º DPO, a dor foi semelhante entre grupos (p = 0,701), com médias de 2,20 (flexores), 2,18 (reto femoral) e 1,78 (patelar) segundo Escala Visual Analógica (EVA). No 15º DPO, as médias foram 1,27, 1,73 e 3,00, respectivamente, mantendo a semelhança estatística (p = 0,149).

**Conclusão:**

O emprego do enxerto de reto femoral apresenta resultado clínico-funcional equivalente aos de tendão patelar e flexores nos primeiros seis meses. ***Nível de Evidência II; Coorte prospectivo.***

## INTRODUCTION

Rupture of the anterior cruciate ligament (ACL) is one of the most frequent and clinically relevant ligamentous injuries of the knee, particularly in young and physically active individuals, being associated with joint instability, functional limitation, and inability to engage in sports.^1^,^2^ In such cases, surgical reconstruction has been established as the treatment of choice to restore stability, allow a safe return to functional and sports activities, and reduce the risk of early joint degeneration.^3,4^

Traditionally, the most commonly used autografts include the patellar tendon and the hamstring tendons, widely validated techniques that are, however, associated with donor-site morbidity, such as anterior knee pain, discomfort when kneeling, and possible specific strength deficits.^3,5,6^ In recent years, the use of the rectus femoris tendon graft, as a variation of the quadriceps tendon graft, has attracted growing interest owing to its favorable biomechanical properties, adequate tensile strength, satisfactory diameter, and potential for lower functional impact on the donor region, with increasing application in combined reconstructions and surgical revisions.^1,4,7,8^

However, despite the expansion of graft options, there is still no definitive consensus regarding the superiority of one graft type over the others in terms of functional outcomes, pain, and stability, and the literature remains divergent when comparing the patellar tendon, hamstring tendons, and quadriceps grafts.^2,5,6^ While some studies associate the patellar graft with a higher incidence of anterior knee pain, others suggest specific strength deficits related to the hamstring tendons, without consistent demonstration of functional superiority among the techniques.^3,6,9^ In this context, it becomes relevant to compare, within the same cohort, the clinical and functional outcomes of ACL reconstruction with rectus femoris tendon, patellar tendon, and hamstring tendon autografts, in order to support a more individualized and evidence-based surgical choice.

## MATERIALS AND METHODS

### Study design

This is a prospective, observational cohort study conducted at a referral university hospital between 2024 and 2025.

### Participants

Patients of both sexes, aged between 18 and 50 years, with complete primary ACL rupture and an indication for surgical reconstruction were included. All procedures were performed by surgeons from the same team, following a standardized technique. Patients with ACL re-rupture, multiligamentous injuries, the need to use more than one graft type, and those who did not complete the planned postoperative follow-up were excluded.

### Study groups

Patients were allocated into three groups according to the graft type used in the reconstruction, namely: rectus femoris tendon graft; patellar tendon graft; and hamstring tendon graft.

The graft choice was defined by individualized clinical criteria, integrating the surgeon's experience and shared decision-making with the patient, while respecting the anatomical and functional particularities of each individual.

### Clinical and functional assessment

The assessments were performed preoperatively and at standardized postoperative follow-up at one day, 15 days, and 6 months after the procedure. Accordingly, criteria such as pain, measured using the VAS,^10^ knee function, assessed by the Lysholm score,^11^ range of motion, measured by goniometry,^12^ and joint stability, assessed by the Lachman, anterior drawer, and pivot shift tests, were analyzed.

### Statistical analysis

Continuous variables were described as mean and standard deviation or median and interquartile range, according to their distribution. Categorical variables were presented as absolute and percentage frequencies. Normality was verified using the Shapiro–Wilk test.

For comparison of baseline characteristics among the graft groups, Pearson's chi-square or Fisher's exact tests were used for categorical variables, and one-way ANOVA or Kruskal–Wallis for continuous variables, according to normality. The evolution of active flexion and extension range of motion of the operated knee was assessed using linear mixed-effects models, considering time and graft type as fixed effects and the patient as a random effect, with analysis of the interaction between graft and time.

When pertinent, pairwise comparisons between grafts were performed at each assessment time point, based on the estimated marginal means, using t-tests with Bonferroni adjustment. The Lysholm score (preoperative and six months) was also analyzed using a linear mixed-effects model with the same effects. Postoperative pain intensity, given its non-normal distribution, was compared between groups using the Kruskal–Wallis test. A 95% confidence interval (CI) was adopted, with a significance level of 5% (α = 0.05). Analyses were performed using R software.

### Ethical aspects

The study was approved by the Research Ethics Committee (opinion No. 7,053,125; CAAE: 82397724.0.0000.5134), and all participants signed the Free and Informed Consent Form.

## RESULTS

A total of 35 patients who underwent anterior cruciate ligament (ACL) reconstruction were evaluated, of whom 15 (42.9%) received a hamstring tendon graft, 11 (31.4%) a rectus femoris graft, and 9 (25.7%) a patellar graft. The mean age of the sample was 31.0 ± 10.9 years, with no statistically significant difference among groups (p = 0.981). A predominance of males was observed (80.0%), with a similar distribution among graft types (p = 0.220).

No statistically significant differences were identified among groups regarding self-reported skin color (p = 0.187), smoking (p = 0.699), alcohol consumption (p = 0.860), or body mass index class

(p = 0.715), demonstrating comparability among the demographic, clinical, and anthropometric characteristics of the sample.

**Figure 1:**
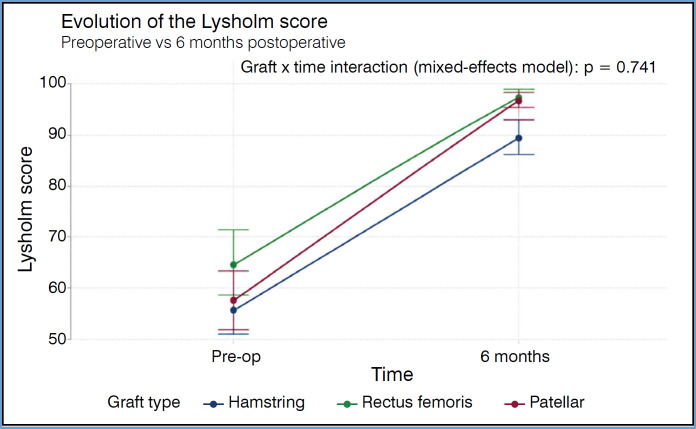
Evolution of the Lysholm functional score from the preoperative period to six months after anterior cruciate ligament reconstruction, according to graft type.

In the preoperative period, the active extension range of motion (ROM) of the operated knee showed medians equal to 0° in all groups, with no statistically significant difference (p = 0.262). Similarly, the preoperative scores of the Lysholm functional scale were similar among groups (p = 0.527), indicating a comparable initial functional condition (Figure 1).

**Figure 2:**
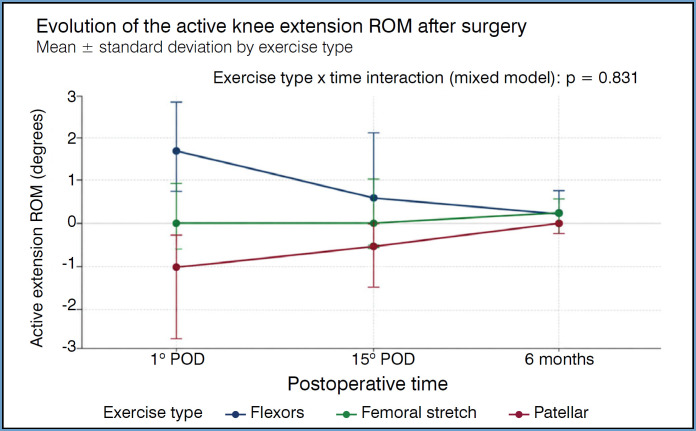
Evolution of the active extension range of motion of the operated knee throughout the postoperative period, according to graft type.

**Figure 3:**
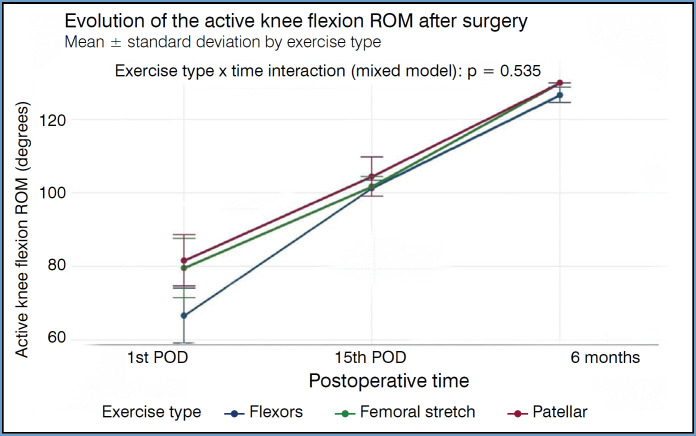
Evolution of the active flexion range of motion of the operated knee throughout the postoperative period, according to graft type.

On the first postoperative day (POD 1), the active flexion ROM showed medians of 70° (interquartile range - IQR: 45°–85°) in the hamstring group, 90° (IQR: 55°–100°) in the rectus femoris group, and 90° (IQR: 85°–90°) in the patellar group. Although the hamstring group presented numerically lower values, the inferential analysis did not demonstrate statistically significant differences among the graft types (p = 0.301). The estimated mean differences were −12.97° (p = 0.164) between hamstring and rectus femoris, and −15.00° (p = 0.110) between hamstring and patellar (Figures 2 and 3).

Regarding active extension on POD 1, all groups maintained medians of 0°, full extension, with no evidence of statistical disparity (p = 0.217). Pairwise comparisons confirmed this similarity, with estimated differences ranging from 1.11° to 2.78°, encompassing the null value in all comparisons. Longitudinal analysis using linear mixed-effects models revealed a progressive and significant recovery of ROM throughout follow-up (time effect: p < 0.001). However, the clinical trajectory was not influenced by the graft type used, as indicated by the absence of a significant graft-by-time interaction for flexion (p = 0.535) and extension (p = 0.831) (Figures 2 and 3).

Analysis of the Lysholm functional scale demonstrated a significant improvement in scores at six months postoperatively in all groups compared with the preoperative period (p < 0.001). However, no statistically significant differences were observed among groups, either in the main effect of the graft (p = 0.167) or in the graft-by-time interaction (p = 0.742).

Postoperative pain intensity, assessed by the VAS (1 to 10), remained low throughout the entire follow-up. On the first postoperative day, mean pain scores were similar among groups (p = 0.701), with values of 2.20 (SD = 1.74) for the hamstring group, 2.18 (SD = 1.40) for the rectus femoris group, and 1.78 (SD = 1.30) for the patellar group. At 15 days postoperatively, a global reduction in pain was observed, with means of 1.27 (SD = 0.59), 1.73 (SD = 1.27), and 3.00 (SD = 3.08), respectively, with no statistically significant differences among groups (p = 0.149). At six months, pain scores remained low and comparable among groups (p = 0.102).

Overall, all groups showed satisfactory clinical and functional progression, with progressive recovery of mobility, significant functional improvement, and low pain intensity, without statistically significant differences related to the graft type used.

## DISCUSSION

Historically, the patellar graft was considered the gold standard for ACL reconstruction, especially in athletes with high functional demand, owing to its high biomechanical strength and the favorable bone integration provided by the bone–tendon–bone model.^1^ However, despite good results in terms of joint stability, its use is associated with a higher incidence of anterior knee pain, discomfort when kneeling, and greater donor-site morbidity, factors that may negatively impact patient satisfaction.^4^

Hamstring tendon grafts came to be widely used as an alternative to the patellar graft precisely because of the lower incidence of anterior knee pain; nevertheless, studies indicate a possible association with specific muscle strength deficits and, in some cases, subtle differences in rotational control, especially in patients with high sports demand.^9^ Thus, although they offer advantages regarding donor-site morbidity, the functional superiority of hamstring tendons over other grafts remains controversial.^9^

The results of the present study, referring to the evaluated period of 0 to 6 months, demonstrate that anterior cruciate ligament (ACL) reconstruction with the rectus femoris tendon graft provides clinical and functional outcomes comparable to those obtained with patellar tendon and hamstring tendon grafts. Consistently, all groups showed significant improvement in knee function and adequate recovery of range of motion, without statistically significant differences among graft types throughout follow-up. These findings reinforce the hypothesis that, from a functional standpoint, the main autografts currently used in ACL reconstruction show similar performance, as suggested by recent literature reviews.^3^

In this scenario, the rectus femoris tendon graft has stood out as a viable alternative, presenting adequate biomechanical properties, good diameter and tensile strength, in addition to good surgical reproducibility.^6^ Studies also indicate lower donor-site morbidity, especially regarding anterior knee pain and difficulty kneeling, when compared with the patellar graft, without compromising joint stability or increasing failure rates.^13^ The findings of the present study are consistent with this perspective, since no significant differences were observed in functional outcomes, recovery of mobility, or postoperative pain intensity among the three graft types evaluated.

The absence of statistically significant differences among groups regarding postoperative pain suggests that, although the literature describes distinct patterns of donor-site morbidity among grafts, these differences do not always translate into relevant clinical impact in the short and medium term.^2,4,14^ In addition, the similar evolution of range of motion, measured by goniometry, and of functional scores indicates that the rehabilitation process was effective regardless of the graft used, corroborating the idea that functional recovery depends not only on the graft type but also on the surgical technique, the rehabilitation protocol, and patient adherence.^14,15^

Among the limitations of this study, the relatively small sample size and the follow-up time limited to six months stand out, which precludes assessing long-term outcomes such as return to sport at the pre-injury level, graft failure, and the development of degenerative knee changes. Previous studies demonstrate that a significant proportion of patients undergoing ACL reconstruction do not return to the same sports level after a few years, regardless of the graft used, which reinforces the importance of investigations with larger samples and prolonged follow-up.^16^

## CONCLUSIONS

The results of this study show that anterior cruciate ligament reconstruction with the rectus femoris tendon graft presents clinical and functional progression similar to that observed with patellar tendon and hamstring tendon grafts. Regardless of the graft type used, patients showed satisfactory functional improvement, adequate recovery of range of motion, and low pain levels during the evaluated period.

Therefore, the choice of graft for ACL reconstruction should be individualized, considering the patient's characteristics, their functional demands, and the surgeon's experience. In light of the findings of this study, the patellar tendon, hamstring tendons, and rectus femoris tendon constitute safe and effective options in the short and medium term. Studies with larger samples and prolonged follow-up are necessary to assess the durability of the results, the return to sports activities, and the long-term safety of the different grafts.

## Data Availability

The contents underlying the research text are available upon reviewers' demand/request.
